# Postpartum Haemorrhage in Canada and France: A Population-Based Comparison

**DOI:** 10.1371/journal.pone.0066882

**Published:** 2013-06-24

**Authors:** Marie-Pierre Bonnet, Olga Basso, Marie-Hélène Bouvier-Colle, Corinne Dupont, René-Charles Rudigoz, Rebecca Fuhrer, Catherine Deneux-Tharaux

**Affiliations:** 1 INSERM, U953, Epidemiological Research Unit on Perinatal Health and Women’s and Children’s Health, Université Pierre et Marie Curie Paris 6, Paris, France; 2 Anaesthesia and Critical Care Department, Hôpital Cochin, Hôpitaux Universitaires Paris Centre, Assistance Publique-Hôpitaux de Paris, Paris, France; 3 Department of Epidemiology, Biostatistics and Occupational Health, McGill University, Montreal, Quebec, Canada; 4 Department of Obstetrics and Gynaecology, McGill University Health Centre, Royal Victoria Hospital, Montreal, Quebec, Canada; 5 Aurore Perinatal Network, Hôpital de la Croix Rousse, Hospices Civiles de Lyon, EA 4129 Université Lyon 1, Lyon, France; Université de Montréal, Canada

## Abstract

**Objective:**

Maternal mortality ratio due to postpartum haemorrhage (PPH) is higher in France than in Canada. We explored this difference by comparing PPH features between these two countries.

**Methods:**

Using data between 2004 and 2006, we compared the incidence, risk factors, causes and use of second-line treatments, of PPH between France (N = 6,660 PPH) and Canada (N = 9,838 PPH). We assessed factors associated with PPH through multivariate logistic models.

**Results:**

PPH incidence, overall (4.8% (95% CI 4.7–4.9) in Canada and 4.5% (95% CI 4.4–4.7) in France), and after vaginal delivery (5.3% (95%CI 5.2–5.4) in Canada and 4.8 (95%CI 4.7–4.9) in France), were significantly higher in Canada than in France, but not after caesarean delivery. Women delivering without PPH were similar between the two populations, except for macrosomia (11% in Canada, 7% in France, p<0.001), caesarean delivery (27% in Canada, 18% in France, p<0.001), and episiotomy (17% in Canada, 34% in France, p<0.001). After vaginal delivery, factors strongly associated with PPH were multiple pregnancy, operative delivery and macrosomia in both populations, and episiotomy only in France (Odds Ratio 1.39 (95% CI 1.23–1.57)). The use of second-line treatments for PPH management was significantly more frequent in France than in Canada after both vaginal and caesarean delivery.

**Conclusion:**

PPH incidence was not higher in France than in Canada and there was no substantial difference in PPH risk factors between the 2 countries. Greater use of second-line treatments in PPH management in France suggests a more frequent failure of first-line treatments and a higher rate of severe PPH, which may be involved in the higher maternal mortality ratio due to PPH.

## Introduction

Postpartum haemorrhage (PPH) remains a major cause of maternal death worldwide [1]–[3], but the maternal mortality ratio due to PPH -defined as the number of maternal deaths due to PPH divided by the total number of live births- differs between countries. In France, this ratio is higher than in other developed countries with comparable national surveillance systems of maternal mortality. In 2004–2006, PPH accounted for 1.40 maternal deaths per 100,000 live births in France [3], compared with 0.66 per 100,000 live births in the United Kingdom [4] and 0.25 per 100,000 live births in Canada (1999–2004) [5]. As death due to PPH is considered largely preventable [3], it is important to understand the reasons for the higher maternal mortality ratio in France. The higher mortality ratio may be explained by a higher PPH incidence, perhaps due to a high prevalence of PPH risk factors in the French population, or by a higher proportion of severe PPH in France, which could result from individual characteristics and/or from inadequacies in PPH first-line treatments [6]. International comparisons are useful to explore these alternatives [7]. Canada and France have a similar level of resources, and available databases make direct comparisons of PPH features between these two countries possible.

The aim of this study was to formally compare PPH incidence, risk factors, causes, and use of second-line treatments in Canada and France, through secondary analysis of data collected in two contemporaneous databases.

## Methods

### Ethic Statement

The Pithagore6 study was approved by the Sud Est III institutional review board and the French Data Protection Authority. The current study was approved by the institutional review board of the Faculty of Medicine of McGill University (IRB Study Number A02-M25-11B). Requirement for informed consent was waived by the ethics committee.

### Data Sources and Study Populations

Data for Canada were obtained from the Discharge Abstract Database (DAD) of the Canadian Institute for Health Information (CIHI), a national database recording all discharges from acute care institutions, and including approximately 98% of all deliveries in each province and territory (except for Quebec) [8]. All medical diagnoses were abstracted from the medical charts by clinicians and were coded, for the period of interest, using the International Classification of Diseases (ICD-10); procedures were coded using the Canadian Classification of Interventions (CCI), supplemented by information routinely collected in the DAD (see Table S1). All hospital deliveries from November 15, 2005 to November 14, 2006 (n = 266,813 deliveries) were identified by a diagnostic code between O10 and O99.8 (with a fifth digit of 01 or 02 indicating delivery) or by a code starting with Z37. We excluded all data from Newfoundland, Prince Edward Island, Nova Scotia, New Brunswick, and British Columbia, as information on parity was unavailable in these provinces (n = 60,842 deliveries). Cases with no mode of delivery specified were also excluded (n = 129). Thus, the Canadian study population included 205,842 deliveries.

The French study population was obtained from the Pithagore6 study [9], a population-based cluster-randomized trial that evaluated a multifaceted educational intervention for reducing the rate of severe PPH in 106 maternity units in 3 regions, comprising 20% of nationwide deliveries. Six perinatal networks were involved in the Pithagore6 trial: the Perinat Centre network around Tours (23 units), the Port-Royal St Vincent de Paul network in Paris (22 units), and the 4 networks of the Rhône-Alpes region: the Aurore network around Lyon (33 units), the Savoie network around Chambery (14 units), the Grenoble network (5 units), and the St-Etienne network (9 units). Data were collected from September 2004 through August 2005 in the Aurore network, and from December 2005 through November 2006 in the other five. All cases of PPH and a 1-to-60 random sample of deliveries without PPH were included. As there was no significant difference between the intervention and reference groups of units with respect to incidence of severe PPH and rates of first- and second-line procedures of PPH management [9], we treated all deliveries included in the Pithagore6 trial as a single population. For each included delivery, a standardized form was used to extract data from medical charts. Mode of delivery was collected for all deliveries recorded in the participating units during the study period.

Delayed and secondary PPH were not included in our study, which focused on primary PPH. In the Canadian database, deliveries with PPH were identified based on the presence of one or more of the following ICD-10 codes: O72.0 (third stage haemorrhage including retained, trapped, or adherent placenta), O72.1 (other immediate postpartum haemorrhage, including haemorrhage following delivery of placenta and atonic postpartum haemorrhage), O72.3 (PPH caused by postpartum coagulation defects), O90.2 (haemorrhage from obstetric wound), R58 (haemorrhage, not classified elsewhere), and T81.0 (haemorrhage complicating a procedure). In order to ensure that the haemorrhage was of obstetric origin, cases identified through the latter three codes were included only if other codes indicating a cause and/or a procedure related to PPH were associated (see codes for PPH causes and interventions in Table S1).

The estimated blood loss used to define PPH was not directly mentioned in the Canadian database; however, the PPH definition proposed by the Society of Obstetricians and Gynaecologists of Canada is a postpartum blood loss of ≥500 mL after a vaginal delivery and of ≥1000 mL after a caesarean delivery [10]. In the French database, the diagnosis of PPH was recorded according to the same definitions of blood loss quantity and based on a clinical assessment of excessive blood loss by the medical staff.

Thus, the Canadian study population consisted of all deliveries for the included provinces (Yukon, Northwest Territories, Nunavut, Alberta, Saskatchewan, Manitoba and Ontario) (n = 205,842), while the French study population consisted of all deliveries with PPH (n = 6,660), and a random sample of deliveries without PPH in the same maternity units (2,414 out of 146,781 deliveries) during the same time period.

### Studied Variables

We examined the characteristics of women, pregnancy, labour, and delivery that had previously been identified as PPH risk factors [11]–[13]: maternal age (<20 years, 20–24, 25–34, and ≥35), parity and previous caesarean delivery combined (primiparous, multiparous without previous caesarean delivery, and multiparous with at least one previous caesarean delivery). Multiple pregnancy, labour induction, regional anaesthesia, operative vaginal delivery, and episiotomy were examined as dichotomous variables. Mode of delivery was examined in 3 categories (spontaneous vaginal delivery, instrumental vaginal delivery, and caesarean delivery), as were gestational age at delivery (<37 completed weeks, 37–41 weeks, >41 weeks), and new-born weight (≤2,500 g, 2,501–3,999 g, ≥4,000 g).

In order to take into account both the pathophysiology of PPH, and the restrictions due to the use of diagnosis codes in the Canadian database (see Table S1), causes of PPH were grouped as follows: uterine rupture or inversion, placenta praevia, coagulopathy, placenta abruptio, trauma (genital tract trauma and surgical trauma), retained placenta, and uterine atony or unidentified cause. Unidentified cause of PPH and uterine atony were classified in the same category. There was no code for PPH from unidentified cause in the Canadian database, but we assumed that the code used for PPH due to uterine atony should have been used in this context, as uterine atony is the most frequent cause of PPH and it is a diagnosis of elimination. Therefore, PPH with unidentified cause are mostly related to undiagnosed uterine atony. In case of PPH due to multiple causes, only one cause was recorded for each case, in the order reported above.

We described second-line treatments in PPH management, including radiologic or surgical procedures needed to control haemorrhage when first-line treatments have failed - arterial embolization, conservative surgical interventions (pelvic vessel ligation and uterine compression suture), and hysterectomy-, transfusion of red blood cells (RBC), fresh frozen plasma (FFP) and platelets, administration of pro-haemostatic agents (fibrinogen concentrates, recombinant activated factor VII, other synthetic coagulation factors, tranexamic acid, anti-thrombin III, aprotinin) and hospitalisation in intensive care unit (ICU), all examined as binary variables (see codes in Table S1).

### Statistical Analysis

We estimated PPH cumulative incidence (with 95% confidence intervals, 95%CI) in the two populations and compared them (χ^2^ test). Cumulative incidence was calculated as the number of PPH cases divided by the total number of deliveries during the examined time period, overall and according to the mode of delivery (vaginal or caesarean).

Among deliveries without PPH, we first described, separately for the French and Canadian populations, the characteristics of the women, pregnancy, labour and delivery, and compared their frequency between the two countries. We then estimated the unadjusted odds ratios (OR) associated with these characteristics for each country, stratifying by vaginal and caesarean delivery. All explanatory variables were included in an unconditional multivariate logistic regression model for each country. Cases with one or more missing values among potential predictors of PPH were not included in the univariate and multivariate analyses (n = 3,506 deliveries, 1.7% of deliveries in the Canadian population, and n = 49 deliveries, 0.54% of deliveries in the French population). However, instances with missing data on birth weight in the Canadian population (n = 16,269 deliveries, 7.90% of total) were included in a separate “missing data” category.

Using the method described by Bruzzi et al. [14], the attributable risk fraction (AR) due to a given predictor was estimated in each population of vaginal deliveries for every factor with an adjusted OR (aOR) significantly different from 1, according to the formula: AR =  (P(aOR-1))/(P (aOR-1) +1), where P is the prevalence of the exposure to the studied predictor in the population of deliveries without PPH, thus assuming that these deliveries would have the same characteristics than deliveries in the general population.

We compared, separately for vaginal and caesarean deliveries, PPH causes between the two countries, as well as the frequency of use of PPH second-line treatments, measured as the number of procedures divided by the total number of vaginal or caesarean deliveries (χ^2^ test). Statistical analyses were performed using STATA Version 11.0 (StataCorp LP, College Station, TX).

## Results

The incidence of PPH was significantly higher in Canada than in France, both overall and among vaginal deliveries, but not among caesarean deliveries (Table 1). Among deliveries without PPH, the distributions of all the examined characteristics of parturients, labour, and delivery were significantly different between the two countries, except for multiple pregnancy and induced labour (Table 2). The largest differences in the studied characteristics concerned the proportion of episiotomy among vaginal deliveries, twice as high in France than in Canada, and the proportions of caesarean delivery and of new-borns with a birth weight ≥4,000 g, higher in Canada than in France.

**Table 1:** PPH cumulative incidence in the French and Canadian populations overall and according to the mode of delivery.

		Canada			France		P^1^
	PPH	All deliveries	PPH incidence	PPH	All deliveries	PPH incidence	
	n (%)	N (% of all deliveries)	(%)^2^ (95% CI)	n (%)	N (% of all deliveries)	(%)^2^ (95% CI)	
**All deliveries**	9,838 (100)	205,842 (100)	4.8 (4.7–4.9)	6,660 (100)	146,781 (100)	4.5 (4.4–4.7)	0.001
**Vaginal deliveries**	7,997 (80.84)	150,636 (73.13)	5.3 (5.2–5.4)	5,627 (84.49)	117,606 (80.12)	4.8 (4.7–4.9)	0.00001
**Caesarean deliveries**	1,841 (18.61)	55,206 (26.80)	3.3 (3.2–3.5)	1,033 (15.51)	29,175 (19.88)	3.5 (3.3–3.8)	0.1168

1 Chi^2^ between France and Canada.

2 PPH per 100 deliveries.

PPH: Postpartum Haemorrhage; CI: Confidence Interval.

**Table 2:** Characteristics of the French and Canadian populations in deliveries without PPH.

	Canada	France	P^a^
	n (%)	n (%)	
Total	196,004	2,413	
Age (years)			<0.001
<20	9,517 (4.9)	29 (1.2)	
20–24	31,948 (16.3)	299 (12.4)	
25–34	119,056 (60.7)	1,611 (66.9)	
≥35	35,483 (18.1)	471 (19.5)	
Parity			<0.001
Primiparous	86,547 (44.2)	1,011 (41.9)	
Multiparous without previous caesarean delivery	85,602 (43.7)	1,147 (47.5)	
Multiparous with previous caesarean delivery	23,855 (12.2)	255 (10.6)	
Multiple pregnancy	3,229 (1.7)	49 (2.1)	0.142
Induced labour	39,332 (20.1)	452 (18.7)	0.104
Regional anaesthesia for delivery	123,724 (63.3)	1.892 (78.5)	<0.001
Mode of delivery			<0.001
Spontaneous vaginal delivery	122,087 (62.3)	1,720 (71.3)	
Operative vaginal delivery	20,552 (10.5)	255 (10.6)	
Caesarean delivery	53,365 (27.2)	438 (18.2)	
Episiotomy	24,840 (17.4*)	662 (33.6*)	<0.001
Gestational age (wk)			0.038
<37	12,824 (6.6)	135 (5.6)	
37–41	158,663 (82.0)	1,977 (81.9)	
>41	21,952 (11.4)	301 (12.5)	
New-born weight (g)			<0.001^b^
≤2,500	8,859 (4.5)	152 (6.3)	
2,501–3,999	149,877 (76.5)	2,095 (86.8)	
≥4,000	21,655 (11.1)	164 (6.8)	
Missing data	15,613 (8.0)	2 (0.08)	

a P for comparison between France and Canada (Chi2).

b P tested without the missing data.

After vaginal delivery, multiple pregnancy and new-born weight ≥4,000 g had the highest adjusted OR for PPH in both populations (Table 3). Other risk factors of PPH after vaginal delivery common to both the French and Canadian populations were: young maternal age, primiparity, previous caesarean delivery, induced labour and operative delivery. Absence of regional anaesthesia for delivery was associated with PPH after vaginal delivery only in Canada, whereas episiotomy and delivery after 41 weeks of amenorrhea were associated with PPH only in the French population.

**Table 3:** Risk factors of PPH after vaginal delivery.

	Canada				France			
	PPH	No PPH	OR	aOR	PPH	No PPH	OR	aOR
	n (%)	n (%)	(95%CI)^a^	(95%CI)^b^	n (%)	n (%)	(95%CI)^a^	(95%CI)^b^
**Total**	7,997	142,639			5,627	1.975		
**Age (years)**								
<20	582 (7.3)	7,950 (5.6)	1.35 (1.24–1.48)	1.19 (1.08–1.30)	120 (2.1)	21 (1.1)	1.99 (1.24–3.18)	1.83 (1.14–2.94)
20–24	1,589(19.9)	25,428 (17.8)	1.14 (1.08–1.21)	1.09 (1.03–1.16)	803 (14.3)	264 (13.4)	1.07 (0.92–1.25)	1.04 (0.89–1.22)
25–34	4,754 (59.5)	86,749 (60.8)	1	1	3,731 (66.4)	1,321 (66.9)	1	1
≥35	1,071(13.4)	22,512 (15.8)	0.86 (0.80–0.92)	0.91 (0.84–0.97)	968 (17.2)	368 (18.6)	0.93 (0.81–1.06)	0.98 (0.85–1.12)
**Parity**								
Primiparous	4,185 (52.3)	61,195(42.9)	1.50 (1.43–1.57)	1.45 (1.37–1.53)	2,912 (51.8)	823 (41.7)	1.59 (1.43–1.77)	1.32 (1.16–1.50)
Multiparous without previous caesarean delivery	3,573 (44.7)	77,016 (54.0)	1	1	2,367 (42.1)	1,070 (54.2)	1	1
Multiparous with previous caesarean delivery	239 (3.0)	4,428 (3.1)	1.14 (0.99–1.31)	1.16 (1.01–1.33)	348 (6.2)	82 (4.2)	1.91 (1.48–2.45)	1.75 (1.35–2.26)
**Multiple pregnancy**	194 (2.4)	1,189 (0.8)	3.10 (2.61–3.69)	3.34 (2.74–4.06)	133 (2.4)	25 (1.3)	1.87 (1.22–2.88)	2.33 (1.47–3.70)
**Induced labour**	2,218 (27.7)	31,161 (21.9)	1.38 (1.31–1.46)	1.34 (1.27–1.42)	1,354 (24.1)	378 (19.1)	1.35 (1.18–1.53)	1.23 (1.07–1.41)
**Regional anaesthesia for delivery**	4,014 (50.2)	74,089 (51.9)	0.97 (0.92–1.02)	0.79 (0.75–0.83)	4,467 (79.4)	1465 (74.2)	1.35 (1.20–1.52)	1.00 (0.88–1.14)
**Episiotomy**	1,607 (20.1)	24,840 (17.4)	1.21 (1.14–1.28)	0.93 (0.87–0.99)	2,651 (47.1)	662 (33.6)	1.76 (1.58–1.96)	1.39 (1.23–1.57)
**Operative vaginal delivery**	1,775 (22.2)	20,552 (14.4)	1.71 (1.62–1.81)	1.62 (1.52–1.72)	1,278 (22.7)	255 (16.6)	1.90 (1.64–2.20)	1.43 (1.22–1.68)
**Gestational age (wk)**								
<37	470 (6.0)	7,961 (5.6)	1.07 (0.97–1.19)	1.10 (0.99–1.24)	266 (4.7)	96 (4.9)	1.03 (0.81–1.31)	1.17 (0.86–1.58)
37–41	6,285 (79.6)	116,425 (82.5)	1	1	4,388 (78.0)	1.635 (82.78)	1	1
>41	1,141 (14.5)	16,745 (11.9)	1.28 (1.20–1.37)	1.04 (0.97–1.11)	973 (17.3)	244 (12.4)	1.49 (1.28–1.73)	1.26 (1.07–1.47)
**New-born weight (g)**								
≤2,500	260 (3.3)	5,399 (3.8)	0.89 (0.78–1.01)	0.79 (0.68–0.92)	235 (4.4)	100 (5.1)	0.87 (0.68–1.11)	0.73 (0.54–1.00)
2,501–3,999	5,820 (72.8)	112,184 (78.7)	1	1	4,784 (85.0)	1,757 (89.0)	1	1
≥4,000	1,287 (16.1)	14,831 (10.4)	1.68 (1.58–1.79)	1.74 (1.63–1.86)	600 (10.7)	117 (5.9)	1.88 (1.53–2.32)	1.90 (1.54–2.34)
Missing data	630 (7.9)	10,225 (7.2)	1.12 (1.03–1.23)	0.92 (0.83–1.02)				

a Simple logistic regression.

b Multivariable logistic regression including all listed variables.

Among potentially modifiable factors, episiotomy had an attributable risk for PPH after vaginal delivery of 11.6% in France, while it was not a risk factor in Canada. Macrosomia, induced labour and operative vaginal delivery had slightly higher attributable risk fractions for PPH after vaginal delivery in Canada than in France (respectively 7.7%, 6.9% and 8.2% in Canada, compared with 5.1%, 4.2% and 5.3% in France) (see Table S2).

Among caesarean deliveries, multiple pregnancy and preterm delivery were PPH risk factors in both countries, whereas new-born weight ≥4,000 g was a risk factor of PPH only in the Canadian population (see Table S3).

Uterine atony (grouped with unidentified cause), was the most frequent cause of PPH after vaginal and caesarean delivery in both populations, but more so in the Canadian one (Tables 4 and S4). In both populations, placental retention and trauma were the second most frequent causes of PPH after vaginal delivery, whereas it was trauma and placenta praevia after caesarean delivery. Among PPH due to trauma after vaginal delivery, 69.0% (n = 696) were related to episiotomy in France, as compared with 35.8% (n = 316) in Canada (p<0.0001).

**Table 4:** Causes of PPH after vaginal delivery.

Causes of PPH	Canada		France		P^a^
	n	%	n	%	
Atony or unidentified	5,655	70.7	2.847	50.6	
Retained placenta	1,277	16.0	1,653	29.4	
Genital tract trauma	871	10.9	1,011	18.0	
Placenta abruptio	116	1.5	17	0.3	
Coagulopathy	44	0.6	18	0.3	
Placenta praevia	13	0.2	71	1.3	
Uterine rupture or inversion	21	0.3	10	0.2	
Total	7,997	100.0	5,627	100.0	<0.0001

Only one cause of PPH was recorded for each case.

a P for comparison between France and Canada (Chi2).

Overall, the use of radiologic or surgical procedures for controlling the bleeding in second-line PPH management was significantly higher in France than in Canada, both after vaginal and caesarean delivery (Tables 5 and S5). Although the rate of hysterectomy did not significantly differ between the two countries, 53% of hysterectomies were performed after an embolization or a conservative surgical procedure in the French population, as opposed to 17% in the Canadian one. FFP transfusion, administration of pro-haemostatic agents, and hospitalization in ICU were also significantly more frequent in France than in Canada.

**Table 5:** Comparison of rates of transfusion, radiologic and surgical procedures and hospitalization in intensive care unit, for PPH management after vaginal delivery between France and Canada.

Procedures	Canada		France		P^a^
	N = 150,636		N = 117,606		
	n	/10000	n	/10000	
Red Blood Cells transfusion	471	31.3	381	32.4	0.606
Fresh Frozen Plasma transfusion	49	3.3	174	14.8	<0.001
Platelets transfusion	25	1.7	36	3.1	0.017
Pro-haemostatic agents	10	0.7	23	2.0	0.003
Radiologic or surgical haemostatic procedures^b^	60	4.0	159	15.5	<0.001
Arterial embolization	19	1.3	116	9.9	<0.001
Conservative surgical interventions	19	1.3	26	2.2	<0.001
Hysterectomy	35	2.3	38	3.2	0.157
Hospitalisation in ICU	84	5.6	149	13.7	<0.001

a P for comparison between France and Canada (Chi2).

b including embolization, conservative surgical interventions (pelvic vessel ligation, uterine compression suture) and hysterectomy.

VD: Vaginal Delivery.

ICU: Intensive Care Unit.

## Discussion

In this study, we found that PPH incidence was significantly higher in Canada than in France overall and after vaginal delivery, even though these differences are not clinically relevant. PPH incidence was not significantly different between France and Canada after caesarean delivery. PPH risk factors were quite similar between France and Canada. However, episiotomy, performed twice as frequently in France as in Canada at the time of the study, was associated with PPH only in France, while macrosomia, twice as frequent in Canada as in France, was more strongly associated with PPH in Canada. Second-line treatments in PPH management were used significantly more frequently in France than in Canada.

International comparisons of PPH features can provide evidence on the role of specific risk factors and clues for prevention. Our comparison between France and Canada is based on data referring to the same time period. An other strength of the present study is its population-based design: all deliveries in 5 provinces and 3 territories in Canada were included, and the French population comprised all deliveries with PPH in a defined geographic area and a sample of control pregnancies, characteristics of which did not differ from the national data [15], thus enhancing the validity of our findings.

In our study, as is common in international comparisons, data were extracted from sources of different nature between Canada and France - respectively a hospital abstract database and a prospective cohort. This difference between the two data sources may have biased our results. However, several considerations make this possibility unlikely. First, in both data sources, PPH diagnosis was a clinical diagnosis, performed by physicians in charge of the parturients. The PPH definition used was the same in both populations, which is also the one recommended internationally [16]. Moreover, in order to harmonize as much as possible the ascertainment of cases between the two countries, ICD10 codes used to extract PPH cases in the Canadian database were expanded to identify cases without the usual codes O72. Second, the prospective design of the French trial may have resulted in better ascertainment of PPH cases in France. But this seems unlikely, as we found a slightly higher incidence of PPH in Canada than in France. Furthermore, the Pithagore6 trial was a cluster-randomized trial. In this trial, the maternity units were randomized, not the parturients, and the intervention was provided at the level of the care providers, not at the patient level. As a result, there was no individual patient monitoring and follow-up in the French trial, and data collection was close to routine practice evaluation, as in the Canadian database. Third, a validation study of perinatal data in the Canadian Discharge Abstract Database reported that most perinatal indicators showed a high degree of accuracy, in particular for PPH, with sensitivity and specificity above 90% and 95%, respectively, as compared to medical files [17]. For the above reasons, we do not think that differences in ascertainment of PPH between France and Canada in our study led to differential identification of cases that would have biased the results we found.

The incidence of PPH was similar in France and Canada, but higher than the estimates reported in recent studies carried out in other developed countries, such as the USA [18] and Ireland [19], where it was <3%. In those two studies conducted on national databases, ICD codes were used to identify cases, but with a narrower definition than in our study, which might explain the difference with our estimates. Conversely, the multi-country incidence of PPH reported in a recent meta-analysis was higher than what we observed, with PPH occurring in 10.8% of deliveries, with wide variations by study and across geographical regions, higher incidences being reported from developing countries [20]. These discrepancies emphasize the importance of using PPH definitions as similar as possible when comparing PPH between countries with similar level of resources, as we attempted to do.

Distinction between emergency and elective caesarean deliveries was not possible in the Canadian data. However, these two modes of delivery differ in terms of risk of PPH, emergency caesarean delivery being associated with higher risk of haemorrhage [21], [22]. Therefore, we consider that our analysis in caesarean delivery is less informative in this context. This is a limit of our study.

In the time period examined, the proportion of vaginal deliveries with episiotomy in France was almost double that of Canada, and episiotomy was a risk factor for PPH in France and not in Canada. This higher proportion of episiotomy in France may be partly related to the lower frequency of caesarean delivery. However, the difference in the proportion of episiotomy was so substantial that it cannot be entirely explained by variations in indications for caesarean section between these two countries. Furthermore, the high rate of episiotomy in France has already been highlighted [23], resulting in the publication of national guidelines in 2006 for limiting its use. Indeed, a recent review demonstrated that applying a policy of restrictive episiotomy has a number of benefits compared with routine episiotomy, including fewer instances of PPH and hematoma [24]. Moreover, this same study showed that the risk of severe perineal trauma was increased with routine use of episiotomy as compared with restrictive episiotomy. Our results, showing that the rate of trauma associated with episiotomy as a cause of PPH was significantly higher in France than in Canada, corroborate these findings. Therefore, a more restrictive use of episiotomy should be considered in France. Following the publication of the above guidelines, the rate of episiotomy has declined in France, but still remains higher than in Canada [15]. Efforts should be continued to decrease the use of episiotomy in France.

Macrosomia was a risk factor for PPH after vaginal delivery in both populations, with a greater impact in the Canadian population, where the proportion of babies weighting ≥4,000 g is significantly higher than in France. Macrosomia is an increasing problem in developed countries, particularly in North America [25], [26]. Its association with maternal high body mass index and diabetes mellitus are now well demonstrated [27], [28], and the higher rate of macrosomia in Canada is probably explained at least in part by the higher prevalence of obesity in North America [29]. The higher risk of PPH associated with macrosomia could be a direct consequence of the new-born’s size or as a result of the increased risks of labour induction, operative vaginal delivery, uterine atony, and perineal tears [30].

We found that second-line treatments in PPH management were performed significantly more frequently in France than in Canada, after both vaginal and caesarean delivery. A recent English national cohort study also reported lower rates of second-line therapies for PPH in United Kingdom than the ones we saw in France [31]. This more frequent use of second-line treatments suggests a greater rate of severe PPH in France. The use of PPH second-line treatments as markers of severity has some limitations, as their performance also depends on clinical practices and resources. However, these procedures are classically considered as such in literature [7], [31], [32], as their use in non-severe PPH remains anecdotal. This greater use of PPH second-line treatments in France may result from a more frequent failure of PPH first-line treatments, which could lead to a higher mortality ratio. Indeed, it has already been demonstrated that inadequate first-line PPH management was involved in PPH severity [6].There is also growing evidence that the use of oxytocin during labour is an independent risk factor of severe PPH [33], [34]. However, we were unable to directly compare the use of oxytocin during labour and first-line PPH management between the two countries, as data on these practices were not available in the Canadian database. Consequently, we cannot further explore the hypothesis that variations in oxytocin use during labour and in first-line PPH management may play a role in differences in PPH severity and, as a consequence, in maternal mortality ratio due to PPH between the two countries. It is well known that promptness is essential in PPH management [6], [35]. The more frequent use of conservative procedures for PPH management in France might induce a delay in hysterectomy, as illustrated by the higher proportion of hysterectomies preceded by conservative surgery and/or embolization in France. This delay in the use of the more radical procedure itself may have an impact on final maternal outcomes.

### Conclusions

In this study, PPH incidence was not higher in France than in Canada. Although PPH risk factors were similar between the two countries, there were some specific differences concerning episiotomy and macrosomia, showing that prevention strategies should be adapted to population characteristics and care practices. Greater use of second-line treatments in PPH management in France suggests a more frequent failure of first-line treatments and a higher rate of severe PPH that may result in a higher maternal mortality due to PPH in this country.

## Supporting Information

Table S1 International Statistical classification of Diseases and related Health Problems, 10^th^ Revision (ICD-10CA) and Canadian Classification of health Interventions (CCI) codes used for identifying diagnosis causes of PPH and procedures in the Canadian discharge Abstract database.
(DOCX)

Table S2 Attributable risk fractions for PPH after vaginal delivery.
(DOCX)

Table S3 PPH risk factors after caesarean delivery.
(DOCX)

Table S4 Causes of PPH after caesarean delivery.
(DOCX)

Table S5 Rates of transfusion, radiologic and surgical procedures and hospitalization in intensive care unit, for PPH management in the context of caesarean delivery.
(DOCX)
