# Supplementary material for: Postpartum Haemorrhage in Canada and France: A Population-Based Comparison
Source: PLoS One. 2013 Jun 24;8(6):e66882. doi: 10.1371/journal.pone.0066882 (PMC3691240; doi:10.1371/journal.pone.0066882)
Supplement: Table S1 — International Statistical classification of Diseases and related Health Problems, 10th Revision (ICD-10CA) and Canadian Classification of health Interventions (CCI) codes used for identifying diagnosis causes of PPH and procedures in the Canadian discharge Abstract database. (DOCX) [file pone.0066882.s001.docx]

**Supporting Table S1**

International Statistical classification of Diseases and related Health Problems, 10^th^ Revision (ICD-10CA) and Canadian Classification of health Interventions (CCI) codes used for identifying diagnosis causes of PPH and procedures in the Canadian discharge Abstract database

| **Diagnosis/procedure code** | **ICD-10CA/CCI** |
| --- | --- |
| Postpartum haemorrhage | O72.0, O72.1, O72.3, O90.2, R58, T81. |
| **Patient, pregnancy, labour and delivery characteristics** | |
| Maternal age | Specific DAD variable |
| Parity | Specific DAD variable |
| Previous caesarean delivery | O34.20, O66.41 O75.70 |
| Multiple pregnancy | Z37.2, Z37.3, Z37.4, Z37.5, Z37.6, Z37.7, O30, O31, O32.5 |
| Labour induction | 5.AC.30, O61 |
| Anaesthesia for delivery | Specific DAD variable |
| Non operative vaginal delivery | 5.MD.50, 5.MD.51, 5.MD.52, 5.MD.56.AA, 5.MD.56NL, 5.MD.56.NM, 5.MD.56. NP, 5.MD.56. NQ, 5.MD.56. NU, 5.MD.56. NV, 5.MD.56. GH, 5.MD.56. PA, 5.MD.56. PB, 5.MD.56. PD, 5.MD.56. PE, 5.MD.56. PG, 5.MD.56. PH |
| Operative vaginal delivery |  |
| With forceps | 5.MD.53, 5.MD.55, 5.MD.56NN, 5.MD.56NR, 5.MD.56NW, 5MD56PC, 5MD56PF, 5.MD.56PJ |
| With vacuum | 5.MD.54, 5.MD.55 |
| Episiotomy | 5MD50GH, 5.MD.53KS, 5MD53JE, 5MD53KL, 5MD53KN, 5MD53KJ, , 5MD53KS, 5.MD.54.KJ, 5.MD.54.KL, 5.MD.54.KN, 5.MD.54.NF, 5.MD.55.KN, 5.MD.55.KL, 5.MD.55.KJ, 5.MD.55.KR, 5.MD.56. GH, 5.MD.56. PA, 5.MD.56. PB, 5.MD.56. PD, 5.MD.56. PE, 5.MD.56. PG, 5.MD.56. PH, 5MD56PC, 5MD56PF, 5.MD.56PJ |
| Caesarean delivery | 5.MD.60 |
| Gestational age | Specific DAD variable |
| Preterm birth | O60.1 |
| Posterm birth | O48 |
| New-born weight | Specific DAD variable |
| **PPH causes** | |
| Retained placenta | O72.0 |
| Atony or unidentified | O72.1 |
| Coagulopathy | O72.3 |
| Placenta abruptio | O45 |
| Placenta praevia | O44 |
| Trauma | O70.2, O70.3, O71.3, O71.4, O71.7, O90.2, Y60.0, T81.0, T81.2 |
| Uterine rupture, uterine inversion | O71.01, O71.11, O71.2 |
| **Interventions** | |
| Blood transfusion | Special CIHI variable |
| Pro-haemostatic agents | 1.ZZ.35.LAC2, 1.ZZ.35.HAC2, 1.ZZ.35.YAC2 |
| Embolisation or pelvic vessels ligation | 1.RM.13, 1.KT.51 |
| Suture of uterus | 5.PC.91.LA |
| Hysterectomy | 5MD60RC, 5MD60RD, 5MD60KE, 5MD60CB, 1.RM.89.LA, 1.RM.89.CA, 1.RM.91.LA, 1.RM.91.CA, 1.RM.87.LAGX |
| Hospitalisation in intensive care unit | Special CIHI variable |
| Maternal death | Special CIHI variable |
