# Supplementary material for: Postpartum Haemorrhage in Canada and France: A Population-Based Comparison
Source: PLoS One. 2013 Jun 24;8(6):e66882. doi: 10.1371/journal.pone.0066882 (PMC3691240; doi:10.1371/journal.pone.0066882)
Supplement: Table S2 — Attributable risk fractions for PPH after vaginal delivery. (DOCX) [file pone.0066882.s002.docx]

**Table S2**: Attributable risk fractions for PPH after vaginal delivery

| PPH Risk factors | Attributable risk fractions (%) | |
| --- | --- | --- |
|  | Canada | France |
| Maternal age<20 | 1.1 | 0.9 |
| Maternal age between 20 and 24 | 1.6 | / |
| Primiparity | 16.2 | 11.8 |
| Multiparity with previous caesarean delivery | 0.5 | 3.1 |
| Multiple pregnancy | 1.8 | 1.7 |
| Induced labour | 6.9 | 4.2 |
| Operative vaginal delivery | 8.2 | 5.3 |
| Episiotomy | / | 11.6 |
| Delivery after 41 weeks of gestation | / | 3.1 |
| New born weight of 4000g or more | 7.7 | 5.1 |
