# Supplementary material for: Postpartum Haemorrhage in Canada and France: A Population-Based Comparison
Source: PLoS One. 2013 Jun 24;8(6):e66882. doi: 10.1371/journal.pone.0066882 (PMC3691240; doi:10.1371/journal.pone.0066882)
Supplement: Table S3 — PPH risk factors after caesarean delivery. (DOCX) [file pone.0066882.s003.docx]

**Supplementary Table S3**: PPH risk factors after caesarean delivery

|  | **Canada** | | | | **France** | | | |
| --- | --- | --- | --- | --- | --- | --- | --- | --- |
|  | **PPH** | **No PPH** | **OR^a^** | **aOR^b^** | **PPH** | **No PPH** | **OR^a^** | **aOR^b^** |
|  | n (%) | n (%) | **(95%CI)** | **(95%CI)** | n (%) | n (%) | **(95%CI)** | **(95%CI)** |
| **Total** | 1,841 | 53,365 |  |  | 1,033 | 438 |  |  |
| **Age (years)** |  |  |  |  |  |  |  |  |
| < 20 | 68 (3.7) | 1,567 (2.9) | 1.28 (0.99-1.64) | 1.05 (0.81-1.36) | 18 (1.7) | 8 (1.8) | 1.03 (0.44-2.39) | 1.02 (0.43-2.44) |
| 20-24 | 200 (10.8) | 6,520 (12.2) | 0.91 (0.78-1.06) | 0.83 (0.71-0.97) | 107 (10.4) | 35 (8.0) | 1.38 (0.92-2.08) | 1.32 (0.87 -2.01) |
| 25-34 | 1,113 (60.5) | 32,307 (60.5) | 1 | 1 | 632 (61.2) | 290 (66.5) | 1 | 1 |
| ≥35 | 460 (25.0) | 12,971 (24.3) | 1.01 (0.91-1.13) | 1.01 (0.95-1.19) | 275 (26.7) | 103 (23.6) | 1.21 (0.93-1.58) | 1.26 (0.95-1.66) |
| **Parity** |  |  |  |  |  |  |  |  |
| Primiparous | 1,016 (55.2) | 25,352 (47.5) | 0.88 (0.78-1.00) | 0.94 (0.83-1.07) | 430 (41.6) | 188 (42.9) | 0.55 (0.41-0.75) | 0.70 (0.50-0.99) |
| Multiparous without previous caesarean delivery | 393 (21.4) | 8,586 (16.1) | 1 | 1 | 321 (31.1) | 77 (17.6) | 1 | 1 |
| Multiparous with previous caesarean delivery | 432 (23.5) | 19,427 (36.4) | 0.49 (0.42-0.56) | 0.62 (0.53-0.72) | 282 (27.3) | 173 (39.5) | 0.39 (0.28-0.53) | 0.47 (0.33-0.67) |
| **Multiple pregnancy** | 167 (9.1) | 2,040 (3.8) | 2.80 (2.33-3.36) | 2.76 (2.11-3.59) | 122 (11.8) | 24(5.5) | 2.30 (1.46-3.63) | 1.68 (1.01-2.86) |
| **Induced labour** | 420 (22.8) | 8,171 (15.3) | 1.67 (1.50-1.88) | 1.43 (1.26-1.62) | 182(17.6) | 74 (16.9) | 1.05 (0.78-1.41) | 0.95 (0.68-1.33) |
| **Regional anaesthesia for delivery** | 1,602 (87.2) | 49,635 (93.3) | 0.50 (0.43-0.58) | 0.54 (0.47-0.63) | 920 (89.1) | 427 (97.5) | 0.21 (0.11-0.41) | 0.25 (0.13-0.47) |
| **Gestational age (wk)** |  |  |  |  |  |  |  |  |
| <37 | 273 (15.3) | 4,863 (9.3) | 1.85 (1.62-2.11) | 1.69 (1.42-2.01) | 266 (4.7) | 96 (4.9) | 2.50 (1.72-3.63) | 2.51 (1.50-4.21) |
| 37-41 | 1,281 (71.6) | 42,238 (80.8) | 1 | 1 | 4,388 (78.0) | 1.635 (82.78) | 1 | 1 |
| > 41 | 234 (13.1) | 5,207 (10.0) | 1.49(1.29-1.71) | 1.01 (0.86-1.18) | 973 (17.3) | 244 (12.4) | 1.11 (0.79-1.56) | 1.06 (0.73-1.53) |
| **New-born weight (g)** |  |  |  |  |  |  |  |  |
| ≤2,500 | 140 (7.6) | 3,460 (6.5) | 1.42 (1.18-1.70) | 0.82 (0.66-1.03) | 170 (16.5) | 52 (11.9) | 1.51 (1.07-2.12) | 0.56 (0.34-0.91) |
| 2,501-3,999 | 1,080 (58.7) | 37,693 (70.6) | 1 | 1 | 746 (72.4) | 338 (77.4) | 1 | 1 |
| ≥4,000 | 375 (20.4) | 6,824 (12.8) | 1.92 (1.70-2.16) | 1.83 (1.62-2.01) | 114 (11.1) | 47 (10.8) | 1.10 (0.76-1.58) | 1.16 (0.80-1.68) |
| Missing data | 246 (13.4) | 5,388 (10.1) | 1.53 (1.31-1.79) | 0.83 (0.67-1.03) |  |  |  |  |

^a^: Simple logistic regression

^b^: Multivariable logistic regression including all variables
