# Supplementary material for: Postpartum Haemorrhage in Canada and France: A Population-Based Comparison
Source: PLoS One. 2013 Jun 24;8(6):e66882. doi: 10.1371/journal.pone.0066882 (PMC3691240; doi:10.1371/journal.pone.0066882)
Supplement: Table S4 — Causes of PPH after caesarean delivery. (DOCX) [file pone.0066882.s004.docx]

**Supplementary Table S4: Causes of PPH after caesarean delivery**

| **Causes of PPH** | **Canada** | | **France** | | **P^a^** |
| --- | --- | --- | --- | --- | --- |
|  | **n** | **%** | **n** | **%** |  |
| Atony or unidentified | 1,288 | 70.0 | 645 | 62.4 |  |
| Retained placenta | 68 | 3.7 | 16 | 1.6 |  |
| Trauma | 243 | 13.2 | 167 | 16.2 |  |
| Placenta abrutio | 75 | 4.1 | 53 | 5.1 | <0.0001 |
| Coagulopathy | 39 | 2.1 | 14 | 1.4 |  |
| Placenta praevia | 111 | 6.0 | 127 | 12.3 |  |
| Uterine rupture or inversion | 17 | 0.9 | 11 | 1.1 |  |
| Total | 1,841 | 100.0 | 1,033 | 100.0 |  |

Only one cause of PPH was recorded for each case.

^a^ : P for comparison between France and Canada (Chi2)
