# Supplementary material for: Postpartum Haemorrhage in Canada and France: A Population-Based Comparison
Source: PLoS One. 2013 Jun 24;8(6):e66882. doi: 10.1371/journal.pone.0066882 (PMC3691240; doi:10.1371/journal.pone.0066882)
Supplement: Table S5 — Rates of transfusion, radiologic and surgical procedures and hospitalization in intensive care unit, for PPH management in the context of caesarean delivery. (DOCX) [file pone.0066882.s005.docx]

**Supplementary Table S5:** Rates of transfusion, radiologic and surgical procedures and hospitalization in intensive care unit, for PPH management in the context of caesarean delivery

| **Procedures** | **Canada** | | **France** | |  |
| --- | --- | --- | --- | --- | --- |
|  | **(n=55,206 caesarean deliveries)** | | **(n=29,175 caesarean deliveries)** | | **P**^a^ |
|  | n | /10000 | n | /10000 |  |
| **Red Blood Cells transfusion** | 351 | 63.6 | 267 | 91.5 | <0.001 |
| **Fresh Frozen Plasma transfusion** | 81 | 14.7 | 122 | 41.8 | <0.001 |
| **Platelets transfusion** | 47 | 8.5 | 27 | 9.3 | 0.729 |
| **Pro-haemostatic agents** | 3 | 0.5 | 13 | 4.5 | <0.001 |
| **Radiologic or surgical haemostatic procedures^b^** | 185 | 33.5 | 137 | 47.0 | 0.003 |
| **Arterial embolization** | 13 | 2.4 | 67 | 23.0 | <0.001 |
| **Conservative surgical interventions** | 102 | 18.5 | 60 | 20.6 | 0.510 |
| **Hysterectomy** | 87 | 15.8 | 34 | 11.7 | 0.134 |
| **Hospitalisation in ICU** | 110 | 19.9 | 149 | 51.1 | <0.001 |

^a^: P for comparison between France and Canada (Chi2)

^b^: including embolization, conservative surgical interventions (pelvic vessel ligation, uterine compression suture) and hysterectomy

ICU: Intensive Care Unit
